# Clinical research mentorship programme (CRMP) for radiation oncology residents in Africa—building capacity through mentoring

**DOI:** 10.3332/ecancer.2021.1210

**Published:** 2021-03-23

**Authors:** Rebecca KS Wong, Verna Vanderpuye, Joel Yarne, Ntokozo Ndlovu, Nwamaka Lasebikan, Ewa Szumacher, Zahra Kassam

**Affiliations:** 1 Princess Margaret Cancer Center, University Health Network; Department of Radiation Oncology, University of Toronto, Toronto M5G 2M9, Ontario, Canada; 2 National Center for Radiotherapy Oncology and Nuclear Medicine, Korle Bu Teaching Hospital, PO Box KB369, Accra, Ghana; 3 Parirenyatwa Radiotherapy Centre, Department of of Oncology, University of Zimbabwe Faculty of Medicine and Health Sciences, Harare, Zimbabwe; 4 University of Nigeria Teaching Hospital, Enugu 01129, Nigeria; 5 Odette Cancer Center, Sunnybrook Health Sciences Center; Department of Radiation Oncology, University of Toronto, Toronto M4N 3M5, Ontario, Canada; 6 Stronach Regional Cancer Center; Department of Radiation Oncology, University of Toronto, Toronto L3Y 2P9, Ontario, Canada

**Keywords:** mentorship, radiation oncology, resident, clinical research

## Abstract

Research skills are mandatory for all oncology residency training programmes. Creating the environment to foster skills and passion can be a challenge in all settings, and a unique challenge in low and middle income countries (LMICs). Tremendous clinical workload places exceptional demand on clinician teachers, research infrastructure and access to research collaborators with diverse methodological skill sets can be limited. International collaborations, and in particular relationship partnerships (Whitehead *et al* ((2018) Acad Med 93 1760–1763)) can be a useful approach to bridge resource gaps and enrich the support available to trainees (Research EoH ((2014) TDR/ESSENCE/2.14)). The Clinical Research Mentorship Programme (CRMP) is a collaborative initiative created by the University of Toronto Department of Radiation Oncology, Princess Margaret Cancer Centre, delivered in collaboration with LMIC radiation oncology residency programmes with the primary goal of enriching the research experience of LMIC oncology trainees. It was inspired by observing a need, an enthusiasm to collaborate and some seed funding that supported the idea. At the heart of the programme is a formalised relationship, a triad, between a LMIC oncology trainee, their local supervisor and a mentor from Toronto. Within the collaborative environment created between the LMIC and high income country (HIC) institutions, enabled by remote learning technologies, a 12-week research methods seminar kick starts a year-long mentorship for the trainee on their research question. The goal is to enrich the quality of the research experience for the trainee, resulting in dissemination of research findings in international conferences and publications. A standard evaluation package is used (Vuple *et al* ((2021) 6 919–928)). In this paper, through a description of our collaboration, we will highlight how a distant mentorship programme was used to enhance clinical research mentorship skills for radiation oncology trainees in Africa. We hope the format we have chosen will continue to demonstrate effectiveness for our trainees, sustainability for our faculty and institutions and will serve as one mechanism to build radiation capacity for LMIC through collaboration, mentorship and research.

## Background

Inequities in health research contribute towards inequities in health [4, 5]. Teaching of research skills is key to future generations of physicians being capable of asking the right questions, and innovating for the future. Indeed, research is mandated for the medical residency curriculum by the Accreditation Council for Graduate medical Education [6, 7], and recommended for radiation oncology training by International Atomic Energy Agency [8]. Creating the environment to foster research skills and passion among trainees can be challenging in all settings, with unique challenges for low and middle income countries (LMICs). Tremendous clinical workload places exceptional demand on clinician teachers, research infrastructure and access to research collaborators with diverse methodological skill sets can be limited. Mentorship is growing as a structured element of professional training programmes. Successfully delivered, it not only results in a positive influence on personal development and career choices, but also research productivity, publication and grant success. International partnerships to provide mentorship can be a useful approach to bridge gaps, and enrich the support available to trainees in LMICs [2].

Princess Margaret Cancer Centre is the largest cancer centre globally under one roof. It is the first cancer hospital in Canada and one of the five clinical sites that calls the Department of Radiation Oncology, University of Toronto her academic home. Each year, approximately 22 radiation oncology residents and 30 fellows are in training here supported by over 70 radiation oncologists across the department. Guided by our mission to prepare radiation medicine leaders for the future [9], and motivated by the call to action in response to the global gap in radiation oncology capacity [10], we imagined the clinical research mentorship programme.

The Clinical Research Mentorship Programme (CRMP) is a collaborative initiative created by the Princess Margaret Cancer Centre, Department of Radiation Oncology, University of Toronto delivered in collaboration with LMIC radiation oncology residency programmes with the primary goal of enriching the research experience of LMIC oncology trainees. It was inspired by observing a need, an enthusiasm to collaborate and some seed funding that supported the idea.

The collaborations are only possible with highly collaborative colleagues, and their trainees, willing to experiment and explore. Our inaugural partner in 2016 was the National Center for Radiotherapy, the premier training centre in Ghana. Our second collaborator in 2018 was the Parirenyatwa Hospital Radiotherapy Centre, the largest tertiary referral cancer treatment centre in Zimbabwe and the University of Zimbabwe clinical training hospital. It is the only institution in the country that has an oncology residency programme and has served as a regional training centre for clinical oncologist since its inception in the early 90s. For 2020, our upcoming cohort will launch in partnership with The Association of Clinical and Radiation Oncologist of Nigeria, the voice of oncologists in Nigeria and their trainees. There are four fully accredited oncology residency programmes in Nigeria with more centres in the process.

In this paper, we will provide a high level overview of a brief history behind the programme, structure of the programme, guidance to mentors, brief description of the past and upcoming evaluation plan, design, our experience to date and sustainability plan for the future. A new component, designed to enhance mentoring skills for the mentors and supervisors, that will be launched and evaluated in 2020–21, and future directions.

## Brief history

In 2015, Dr Joel Yarney (JY) joined Princess Margaret as a visiting fellow. This sets the stage for Dr Rebecca Wong (RW) visiting Ghana for the first time, as a speaker at the African Radiation Oncology Group meeting. Here the opportunity to meet with many of their residents planted the idea and inspired the programme. Supported for the first 2 years by a grant from the Princess Margaret Cancer Centre Capacity Building Fund, the first year long clinical research mentorship programme was offered in 2016 supporting two residents from the Korle Bu Cancer Hospital in Ghana. This was followed in 2018 where the programme was offered to two residents at the Parirenyatwa Group of Hospitals in Harare, Zimbabwe. In 2020, we plan to support a new cohort of participants from Nigeria through a collaboration with the Association of Radiation and Clinical Oncologists of Nigeria. We invite a new programme each year to collaborate, hoping to learn, through a standardised evaluation strategy, how to adapt to different collaborating environments and collaborating needs and styles, while growing the network, and its capacity to pass on the philosophy.

### The structure of the programme

Details of the training programme, from how the resident research projects were evaluated, selected, programme implementation, evaluation, evaluation metrics and the lessons learned from the first year has been published [3].

**Figure 1.**
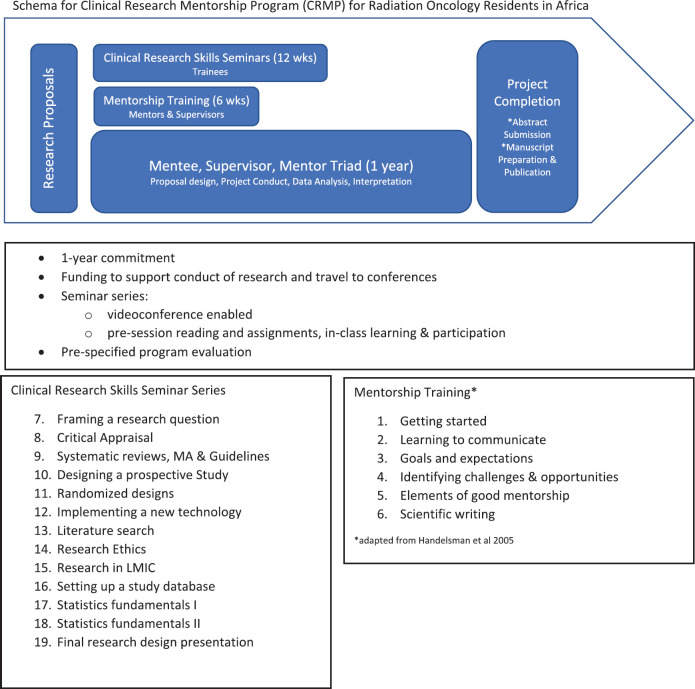
Schema for CRMP for radiation oncology residents in Africa.

In brief, the clinical research mentorship programme is a 1-year programme with four key design features (Figure 1). First, the programme starts with a call for research proposal (2–3 pages). Potential participants are invited to submit a brief research proposal in writing and identify a local research supervisor. The proposals are evaluated based on pre-defined criteria, including relevance of the clinical question, considerations given to the research plan and feasibility of the proposal. Only selected mentees were assigned a mentor at each iteration, due to the pilot nature of the project and faculty resource limitations. Trainees who are not selected are provided written feedback for improvement of their study.

Second, all trainees, whether mentors were assigned or not, are invited to participate in the 10-week seminar series and encouraged to complete their projects with their supervisors. The seminar series is designed to cover a different topic each week from ‘asking the right question’ to setting up a database to research ethics. Sessions employ a flipped classroom approach and use the trainees’ research questions to illustrate the application of the topic. All seminars are delivered using videoconferencing technologies, and informal discussions using predominantly emails.

**Figure 2.**
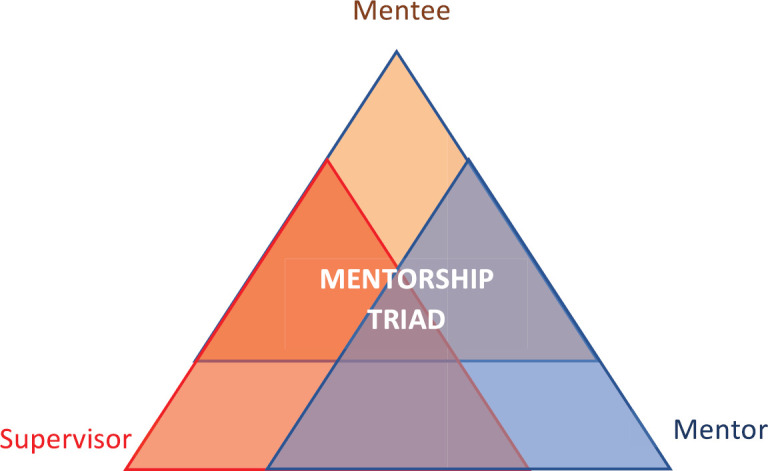
Mentorship triad.

Third, the mentee, supervisor and mentor triad is at the core of the mentorship programme (Figure 2). The supervisor’s role is to provide clinical expertise on the research topic, facilitate problem solving from a systems perspective and played a central role in ensuring the success of the international collaboration. The supervisor is identified by the resident at the time of the research proposal application, and has agreed to take on supervisory responsibilities for the local training programme. The mentors are volunteers from Toronto selected based on their research experience (radiation oncologists with MSc in clinical epidemiology as well as practical experience in conducting and completing research), experience working with our own residents and fellows in Toronto and a passion for teaching and global health. The mentee and supervisor work with the trainee over the course of the year with the goal of supporting the trainee along the research journey from refining the research question to preparation of abstract for submission to an international conference, and manuscript preparation to publication. It is expected all participants within the mentorship triad will benefit through mutual learning.

Fourth, a modest stipend is made available to support in part their travel to a conference to present their results and conduct their research.

### Guidance to mentors

Mentors assigned to the trainees are selected for their scientific expertise, experience as teachers and mentors for trainees entering into clinical research, and their interest and willingness to work with colleagues with our said objectives. Mentors see themselves as invited guests by our host faculty [1], providing suggestions that need to be considered and adapted by the trainee and their local supervisors, serving as a guide to navigate conferences, prepare reports and abstracts and serve as the host during conference visits. To date, no formal mentorship training has been provided, but guided by the key principles of collaboration. For our next iteration, a mentor and supervisor seminar is planned. We will adapt from the ‘Entering Mentoring’ seminar [17, 18], adapting the content to our clinical context. Through a facilitator, supervisors and mentors will engage in discussions ranging from What is a good project? Mentoring philosophy, setting goals and expectations, identifying challenges, elements of good mentoring and what makes good scientific writing. These will be delivered in six 1-hour sessions, planned to start at the beginning (Appendix).

### Evaluation plan

Prior to the start of the programme, an evaluation plan was envisioned. We hypothesise that by using a consistent approach to evaluate learnings from each collaboration, each environment, will be accumulative, translating into greater efficiency and effectiveness with each offering. The evaluation programme consists of pre-programme discussions with our collaborators (and trainees) to understand the learning goals, evaluation surveys post seminars, use of a validated tool to measure critical appraisal skills [15] and post course discussions to elicit their feedback. Qualitative study strategies are used to understand the barriers, enablers and longer term impact through the eyes of the learners. For the 2020 class, we plan to incorporate tools to capture the value of mentoring as introduced below [16].

### Sustainability plan

The programme is designed to enhance skills development in clinical research methods through collaboration. In addition to willingness for all participants to find the time to participate, resources are needed in terms of infrastructure and human resources. These include faculty time for teaching, mentoring, videoconferencing tool subscription, project coordination, evaluation and stipend for knowledge dissemination for the trainee. Outcome metrics are critical for sustainability of the programme. They not only serve to guide improvement of the programme, but are also critical in sustaining collaborator interest, mentees, supervisors and mentor alike, and to support grant applications for funding support.

## Results

Due to limitations in funding, faculty commitment and the pilot nature of the project, two mentees were paired with a mentor at each iteration to date. Seven residents submitted proposals in 2016, and only the two senior residents were selected by their programme to submit proposals in 2018. All mentees who participated in the 2016, and 2018 from Ghana [11] and Zimbabwe [12, 13] have completed their projects and their results were presented at the Canadian Association of Radiation Oncologist Annual Meeting although only one mentee attended the conference in person (Canada, Halifax) due to visa issues.

Early programme evaluation (from Ghana trainees) suggests a high level of engagement and effective learning of critical appraisal and clinical research skills. One manuscript has been published [14] and one manuscript has been submitted [13]. Areas for improvement were also identified and described previously[3].

A qualitative study designed to understand the reasons for participation, other experience in mentorship programmes, perception of the programme, enablers and barriers, suggestions for improvement. Past programme applicants, mentees, supervisors, programme directors and mentors are invited to participate. The study is currently ongoing. Initial findings from this study suggest there were strong engagement by all the participants, the program had enriched the research experience of participating residents, and aligns well with the mentees' formal residency training program objectives. Suggestions for the future include a call for the program to support more trainees within each training programs as well as across more training programs. This will allow communities to emerge that will support each other, grow future generation of mentors and ultimately research capacity.

## Discussion

The concept of mentorship is steeped in tradition with the earliest reference to a mentor figure in the literature appearing in Homer's ‘The Odyssey’ in the 15th century in the form of the character called ‘Mentor’. It means a trusted friend, counsellor or teacher, an experienced adviser and supporter = somebody, more experienced, who advises and guides a younger, less experienced person. What it means, looks like, when does it begin and end, expectations and perceived effectiveness vary widely. Many different models of mentorship have been described. The most commonly cited models are the traditional dyad mode (i.e. one on one mentoring), peer and facilitated peer mentoring, group mentorship [19] and network mentorship [18, 20]. Smabunjak *et al* [21] provided a systematic review on mentoring in academic medicine and found successful mentorship occurs, it is often influential to personal development, career guidance, choice and research productivity, including publication and grant success. Choi *et al* [22] provided a compelling argument on why mentoring need to be a strategic priority for academic medical centres in order to fulfil its missions, and provided valuable suggestions, through merging a top-down and grassroots approaches, to build and sustain a culture of mentorship.

How to create an effective mentorship relationship is a subject of many scholarly works. Mismatch in perception of what is mentorship between mentors and mentees has been described [23, 24] and would benefit from proactive planning and communication. The distinction between a supervisor and a mentor maybe subtle but warrants articulating. One way of operationalising this is around the evaluation function. The supervisor sets expectations, provide evaluation and typically has a responsibility to the institution (e.g. to recommend the best resident within the training programme as a faculty candidate). A mentee often benefits from having many different mentors, with different expertise and perspectives, for different aspects of their lives, at different times of their career.

Finding a good research topic is often not easy and represents an important barrier to scholarly productivity for learners. The difficulty in landing with a good research question contrasts with the belief by education programme leaders that research possibilities in busy clinical environments are abundant [24]. Asking the right question is the cornerstone of any successful project [25]. One of the key roles of mentors and supervisors is to help the trainee select the topic that is at once of interest to the learner, but can also have a realistic prospect on feasibility and barriers. Scholarly productivity including synthesis and interpretation of the data, and presentation in a peer reviewed forum (e.g. international conferences) is an important outcome that should be achievable for most well designed projects. Publication of results requires a combination of topic selection, ability to execute the research plan, research findings, writing and publication strategy and perseverance. Sharing of expectations between mentors, supervisors and learner is a good starting point for a successful relationship.

Successful mentoring can be learned. Pfund *et al* [26] conducted a randomised trial comparing an 8-hour curriculum with no formal intervention with effectiveness demonstrated by improving self-reported competency on a validated questionnaire. The intervention focuses on six key competencies including communication, expectations, assessing mentee’s understanding of scientific research, diversity within mentoring relationships, fostering mentee’s independence and promoting mentee’s professional career development. Henry-Neol *et al* [27] described the importance of structure that pays attention to enunciating the roles of mentors and mentees, expected benefits, communication about the relationship, in addition to ‘soft skills’ such as self-awareness, focus, mutual respect.

How can it be learned? Many scholarly works, courses , handbooks [28] and institutional [29] and society guidelines [30] are available. The International Cancer Expert Corps has a well articulated mentoring structure pairing institutions on mentoring on a broad scope of cancer systems activities [31]. The African Organization Research Training in Cancer with its international members and formal relationships with collaborative partners provides a core network encouraging a meeting of the minds and international mentorships [32]. Often awareness is the first step in developing expertise in mentoring. This is followed by testing and refining over time. Attention and awareness to different learning communication styles that may exist between senior faculty and trainees (who are usually younger) is worth noting [33]. At the individual mentee and mentor interface, worksheets that prompt documentation of key activities such as goals of the relationship, expectations, timelines and planned interactions serve as sign posts that the relationship is active and gives recognition to the mutual commitment to the relationship. Formalised workshops provide additional structure to the process. For the next phase of our project, our trainees–supervisors–mentors triad will be from multiple institutions resulting in a new layer of complexity. We plan to incorporate a mentorship seminar series adapted from ‘entering mentorship’ [17] to facilitate dialogue on mentorship.

We selected a 1-year mentorship programme format allowing mentors, in collaboration with the supervisors, to support their trainee through the course of their research project. This format has the advantage of supporting trainees who are earlier on in their research career where research problem solving skills require nurturing. This format allows the mentor to gain a better understanding of the challenges and enablers of the trainee within the learning environment and contribute to problem solving in ways that more focused programmes will be less adapted to respond. Indeed, the short intense research methods courses [34, 35] and the longitudinal format can be complimentary. The bidirectional learning provides a valuable window for international mentors to learn about the nuances of practicing oncology and the right questions to ask in a setting totally different from their own.

Our early experience supported our hypothesis that a distant mentorship relationship and a local supervisor–distant mentor–mentee triad are efficacious. Despite limited Internet connectivity and technical challenges at times, the clinical research mentorship programme enriched the learners’ experiences as it did, also for the mentors and supervisors. The collaborative spirit for the participants continues to expand beyond the year, with a growing network of CRMP alumni and potentially, future mentors in the making.

## Conclusion

The clinical research mentorship programme format is entering into its third offering. Early success encouraged us to broaden our collaborations and expand our structure into encouraging the development of mentorship skills through a short, structured curriculum. We hope the format we have chosen will continue to demonstrate effectiveness for our trainees, be sustainable for our collaborating faculty and serve to enrich radiation capacity building through collaborative mentorship and research.

## Conflicts of interest

The authors have no conflicts of interest to declare relevant to the content of this manuscript.

## Appendix. CRMP mentor seminar.

### Background

The CRMP is entering into its third collaboration in 2020. Following each iteration, lessons learned is incorporated into the design of the next while focusing on the core objective of providing complimentary advice to the trainee’s own residency programme, supervisor’s research objectives and deliverables. Together with the Nigeria Association of Clinical Oncologist, this offering is expected to involve trainees and supervisors from multiple institutions. There is a desire and opportunity to provide a shared approach to mentorship. The Seminar ‘Entering Mentoring’ by Handelsman *et al* [17] has been adapted and shown effective in a randomised setting. CRMP will adapt the curriculum and invite all mentors and supervisors to participate.

### Format

Six 1-hour discussions conducted via videoconferencing. A facilitator will be assigned to each session.

Assignments designed to encourage reflection and engagement (approx. 1 hour) between session will be provided.

Session will take place prior to the beginning of the programme launch, while the remaining sessions will be scheduled at a time that is convenient for most participants during the first 12 weeks of the programme.

### Curriculum

**Table:**

Session	Topic	At the end of the session, the participant will gain insight into	Suggested activities with your mentee, or for yourself
I	Getting started	What is a good project? Getting to know your mentee/trainee	
II	Learning to communicate	What is a mentoring philosophy and what is ours? What do you expect from your mentee/trainee, and what do they expect from you? How do you deal with different work and learning styles? Tips on effective communications through virtual platforms	Write down our mentoring philosophy
III	Goals and expectations	How do you know if your mentee/trainee understands what you are saying? How do you encourage your mentee/trainee to develop independence?	Document a summary of expectations, timelines
IV	Identifying challenges	How do you know there are problems? How do you know things are going well? How do you provide effective feedback and build confidence in your trainee?	
V	Elements of good mentoring	What has proven effective in your mentoring? What is an effective way of sharing ideas, challenges and learn from other mentors?	Have a bi-directional feedback session with your mentee
VI	Righting writing	What is a good piece of scientific writing? What is the best way of providing feedback to your mentee’s writing?	
